# 纸基微流控芯片在病原体检测中的研究进展

**DOI:** 10.3724/SP.J.1123.2025.04011

**Published:** 2025-11-08

**Authors:** Xintong LIU, Jia SHI, Meng SHI

**Affiliations:** 辽宁师范大学，辽宁 大连 116029; Liaoning Normal University，Dalian 116029，China

**Keywords:** 纸基微流控, 病原体, 纸基材料, 制作方法, 即时检测, paper-based microfluidics, pathogens, paper-based materials, fabrication methods, point-of-care testing

## Abstract

在全球公共卫生形势日益严峻的当下，由细菌、病毒所导致的大范围疾病传播已经成为全球公共卫生领域的一大挑战，严重威胁着人类健康。因此，病原体的快速、准确检测对于预防和控制传染病的传播、保障公共卫生安全具有至关重要的作用。在此背景下，开发高效、简便、成本低廉且可广泛使用的检测方法已成为公共卫生领域的重点研究方向。纸基微流控芯片作为一种新型的检测平台，凭借其独特的集成化设计与特性，为病原体检测开辟了新路径。该装置集成了多种生物识别分子，包括抗体、核酸适配体、噬菌体和酶等，可高效捕获和检测病原体；因其具有低成本、易于加工、生物降解、高热稳定等优势，在医学诊断、环境监测和生化分析等多个领域展现出巨大的应用潜力，尤其是在病原体的即时检测领域，已逐渐成为研究和应用的热点。本文对纸基微流控装置从纸芯片的材料、二维和三维结构设计、制作方法以及检测技术等多个方面进行了详细阐述。同时，本文通过大肠杆菌（*E. coli*）和诺如病毒等的检测案例，展现了纸基微流控装置在病原体快速检测中的显著优势。随着材料科学、纳米技术和生物工程的交叉融合，纸基微流控技术可与高灵敏度传感器、智能化数据处理模块等结合，在病原体检测领域发挥重要作用，实现更精准、更快速的现场诊断，为全球公共卫生安全提供关键技术支撑。

## Abstract (en)

In the context of an increasingly severe global public health situation， diseases caused by bacteria and viruses have become a global threat， severely impacting human health worldwide. Rapid and accurate pathogen detection is crucial for preventing infectious diseases and plays a vital role in safeguarding public health through effective preventive measures. Against this backdrop， the development of efficient， simple， low-cost and widely applicable detection methods has become a key research direction in public health. As a novel detection platform， paper-based microfluidic devices （μPADs） open new pathways for pathogen detection due to their unique design and integrated properties， showing significant potential in medical diagnostics and beyond. Their low cost and ease of use make them particularly appealing for point-of-care pathogen testing， a field in which they are gaining increasing attention. These devices are user-friendly， require no complex equipment， and are easy to transport and store. This article provides an in-depth exploration of μPADs， discussing comprehensive material design considerations for microfluidic applications. It will also examine advanced fabrication techniques and innovative detection technologies in detail. Furthermore， practical detection cases for specific pathogens such as *Escherichia coli （E. coli）* and norovirus will be presented to demonstrate the significant advantages of these devices in applications. As scientific research advances， μPADs are expected to play an increasingly significant role and poised to greatly enhance global public health efforts. Their inherent simplicity and affordability make them ideally suited for regions with limited access to sophisticated laboratory infrastructure. They enable convenient on-site testing， which significantly reduces both time expenditure and operational costs. Furthermore， their portability makes them suitable for deployment in disaster relief scenarios and remote fieldwork. Rapid pathogen identification is crucial for effective outbreak containment. To advance this goal， μPADs can be integrated with high-sensitivity sensors and intelligent data processing modules. Advancements in material science have facilitated the development of highly sensitive and stable paper-based substrates for diverse applications. Simultaneously， nanotechnology enables the fabrication of miniaturized components. Meanwhile， bioengineering has contributed to highly sensitive detection probes. These probes can detect pathogens even at very low concentrations. In conclusion， continuous technological advancements are positioning μPADs at the forefront of diagnostic innovation. As research and development progresses， their role in global public health is anticipated to expand considerably. This progress offers considerable promise for enhancing global disease surveillance and response capabilities. The potential is largely due to the inherent simplicity of μPADs， which makes them accessible worldwide. Their low cost， portability， and ease of operation by personnel with minimal training make them particularly advantageous in low-resource settings and provide a robust platform for rapid， on-site diagnostic testing. The modular design of these devices facilitates extensive customization and scalability. This enables them to be tailored for the detection of a broad spectrum of pathogens. The technology is also readily scalable for mass production， facilitating widespread distribution. These attributes collectively make it a versatile and indispensable tool for global health initiatives. The integration of these devices with mobile technology represents a significant leap forward， enabling real-time digital tracking and monitoring of disease outbreaks. This capability significantly enhances rapid response and epidemic containment efforts. Furthermore， the collected data can also be leveraged for broader public health surveillance and epidemiological studies. The potential of this technology to revolutionize point-of-care diagnostics is immense. μPADs can deliver accurate results within minutes， a stark contrast to the hours or days required by conventional laboratory methods. This rapid turnaround time facilitates faster clinical decision-making and intervention， ultimately leading to improved patient outcomes. Moreover， μPADs hold considerable promise for the rapid detection of novel or emerging pathogens. As research and development continue unabated， the functional capabilities of μPADs are anticipated to expand significantly. Future iterations may incorporate cutting-edge detection methodologies and advancements in nanotechnology to further enhance sensitivity and accuracy. Such enhancements will further solidify the pivotal role of μPADs in global public health surveillance and infectious disease control strategies. Ultimately， μPADs hold immense potential to save lives worldwide by enabling rapid pathogen detection and effectively curbing the transmission of infectious diseases.

病原体的快速传播严重威胁人类健康和公共卫生。由病原体引起的传染病死亡率高、变异率高、危害范围广，不仅给全球经济带来重创，还对公共卫生造成难以估量的损害。病原体可借助水、空气、土壤等媒介直接传播至人类，或通过污染农林牧副渔产品间接危害健康，同时对相关产业造成经济影响，因此，开发即时检测（point-of-care testing，POCT）方法至关重要^［1］^。传统的病原体检测方法，如细菌培养、聚合酶链式反应（PCR）技术、酶联免疫吸附试验（ELISA）等，存在检测时间长、仪器要求高、主观性强、特异性和灵敏度低等缺点。研究人员因此开发了微型PCR技术、基于成簇规律间隔短回文重复序列的即时检验（CRISPR-based POCT）和纳米传感器等技术，以便在有限的环境中实现快速、高灵敏检测，但过高的成本和技术限制了其广泛应用。近年来，多种新型检测方法应运而生，其中基于纸材料的纸基微流控技术作为一种新兴技术脱颖而出。

纸基微流控芯片（microfluidic paper-based analytical devices， μPAD）是通过纸的毛细作用，实现样品预处理、分离和检测的集成化分析^［2］^，在成本、便捷及环保等方面具有显著优势^［3，4］^。μPAD的发展历程可以追溯到19世纪初的石蕊试纸，而1949年纸上薄层色谱技术的出现，为纸基分析方法的发展奠定了坚实基础。20世纪50年代，随着半导体微加工技术的成熟，微流控技术逐渐兴起。2007年，Martinez等^［5］^通过光刻法首次成功制作纸芯片，并提出了μPAD的概念，这标志着现代μPAD的正式诞生，同年蜡印技术开始成为μPAD制造的主要方法。2008年，该团队进一步开发出多层平面纸芯片叠加技术，制成了首个三维纸芯片^［5］^。2009年，电化学检测技术被引入纸芯片领域，有效解决了比色法灵敏度不足的问题^［6］^。此后，折叠法等新型制造技术的出现，让纸芯片的操作变得更加便捷，形式也更加丰富多样。凭借其便携、低成本、一次性使用等显著优势，μPAD在医疗诊断、环境监测、食品安全等诸多领域获得了广泛应用。近年来，μPAD在检测技术以及生物医学诊断等领域的研究持续深化，其应用前景也越来越广阔。目前，该领域主要在纸基表面修饰^［7］^、结构设计、制作方法和检测技术等方面进行不断创新，以提高对病原体的检测能力。本文系统地阐述了μPAD检测病原体的研究进展，分析了不同类型μPAD的优缺点，并对该技术的发展进行了展望。

## 1 μPAD的材料、结构及制作方法

### 1.1 纸材料

#### 1.1.1 纤维素基纸

纸张主要由纤维素构成。纤维素是成本最低且分布最广的绿色原料，也是应用最广泛的纸基材料，其由带有高密度羟基官能团（-OH）和少量羧酸基（-COOH）的纤维制成^［8］^。与硅、玻璃等传统材料相比，纤维素基纸成本低，是可再生资源，其轻便和易折叠使μPAD可微型化和便携化。此外，纤维素基纸具有良好的生物兼容性，能固定酶、DNA和蛋白质等生物分子。其浅背景色有利于进行比色分析，提高检测准确性。常见的纤维素基纸有Whatman滤纸、Filter King滤纸等，可以实现定性和定量分析。Reboud团队^［9］^开发了一种等温扩增的3D μPAD，用于检测疟原虫DNA。该技术以滤纸为核心载体，通过热蜡打印在滤纸上构建疏水通道，经折叠形成多层微流控单元，可完成全血样本的自动化处理。检出限达10^5^ IU/mL，其灵敏度与实验室PCR相当。然而，纤维素基纸存在一些局限性，如机械强度不足、易变色、不耐腐蚀等，这些因素会干扰检测准确性，因此，科研人员不断进行纤维素基纸的化学修饰和改性，增强其性能，使其适应复杂环境，在病原体检测中发挥更广泛的作用。

#### 1.1.2 硝化纤维素膜（nitrocellulose， NC）

NC膜是将纤维素分子中的-OH替换为硝基（-NO_2_）形成的改性材料。基于原材料的差异可分为木浆、棉纤维^［10，11］^、微晶纤维素^［12］^、芒草纤维素^［13］^、细菌纤维素^［13］^等。NC膜是一种多孔亲水性材料，允许液体在膜中自由流动，且对生物分子有较高的非特异吸附，能够固定DNA、酶以及蛋白质等生物大分子，提高检测的特异性和灵敏度，因此，其在μPAD检测病原体中具有良好的应用前景。NC膜的孔径、切割和塑形的多样化，提高了μPAD检测病原体的灵敏度、速度等^［14，15］^。商业化NC膜的孔隙及结构可控，可进行大范围推广。Jain等^［16］^开发了基于NC膜的挂锁探针滚动圈扩增横向流动检测技术，实现了核酸可视化检测。该技术对SARS-CoV-2的LOD为1.78×10⁵ copies/mL，可在唾液、植物提取物等复杂基质中稳定运行。NC膜在病原体μPAD检测中优势显著，然而，要实现其良好应用，需满足一系列前提条件，其中构建疏水屏障至关重要；疏水屏障可精确控制液体流动，防止样品扩散，从而提升检测的准确性与可靠性；该结构可采用蜡、聚二甲基硅氧烷（PDMS）、光刻胶或聚氨酯丙烯酸酯（PUA）等疏水材料，通过物理或化学方式构建，以满足μPAD病原体检测的多样化需求。

#### 1.1.3 纤维素基复合材料

纤维素基复合材料是将纤维素作为基体材料，通过物理、化学方法，与有机或无机材料合成的新型纸基材料，既具备纤维素的优良特性，又引入了新功能，可提高检测特异性和灵敏度，研究人员通过对纤维素基纸进行处理，满足了利用μPAD进行多样化病原体检测的需求，但由于物理化学方法繁多，制作工艺复杂、成本高，使得其在商业化应用中受到限制^［17，18］^。

### 1.2 **μ**PAD结构

纸芯片结构有两种结构（如表1）：二维（2D）和三维（3D），不同结构各有其特点和应用场景。其中2D纸芯片分为两类，第一类为侧向层析试纸条（lateral flow assay strip，LFA），通过在基底材料上构建检测线（T线）和反应线（C线），对靶标分子进行检测，是最常见的快速检测工具之一。Xu等^［19］^设计的新型LFA，通过优化抗原抗体配比和信号放大策略，实现了对多种人乳头瘤病毒（HPV）的高效检测，其中HPV6、11和16型的检出限低至10 copies/μL，显著提升了检测灵敏度，为疾病早期筛查提供了新方案。

**表1:** 纸基结构对比

Type	Structure	Analysis methods	Features
2D	type 1： lateral flow assay strip，a fluid analysis platform based on porous membranes/paper substrates type 2： single-layer planar structure with linearly arranged functional regions	relies on specific antigen-antibody binding or biochemical reactions， with detection achieved through marker signals， immunoanalysis （e.g.， immunochromatography）	limited space and limited multi-target detection capability
3D	three-dimensional channel network formed by folding/bending 2D structures or bonding multi-layer paper substrates	immunoanalysis， biochemical analysis （e.g.， enzymatic reactions， multi-step separation-detection integration）	sample space allows for multi-step operations and is also suitable for multi-target analysis

2D： two-dimensional； 3D： three-dimensional.

第二类2D纸芯片由单层纸构成，通过设计精确的图案，并在纸基上创建疏水屏障，实现流体的精准控制。如Davidson等^［20］^开发了基于环介导等温扩增和比色法的2D μPAD，实现了对唾液中SARS-CoV-2核酸的可视化检测。该技术对SARS-CoV-2的LOD为200 copies/μL，特异性100%，全程耗时小于1 h。此外，多样的纸基材料及其表面丰富的化学修饰可能性，使其能够实现多靶标分子同时检测，目前商品化检测试纸多采用该结构，在生物化学检测领域展现出巨大潜力。

3D纸芯片则是在2D纸芯片的基础上，通过折叠、弯曲、旋转或多层衔接形成的立体结构。Seok等^［21］^开发了基于逆转录环介导等温扩增和荧光检测的全集成3D μPAD，可同时实现人血清中寨卡病毒（Zika virus，ZIKV）、登革热病毒（Dengue virus， DENV）和基孔肯雅病毒的多重检测。该技术以层析纸为基底，通过蜡印疏水屏障构建2D功能分区，并引入3D聚醚砜（PES）不对称膜层叠结构调控垂直流动，其在1 h内可完成血清样本中3种病毒的同步检测，临床样本验证准确率100%。与2D纸芯片相比，3D纸芯片在减小μPAD体积的同时，可完成样品预处理，实现多路检测和不同区域的多流体操纵^［22］^。如在预处理区域实现病原体的捕获、裂解、靶标分子的纯化与富集，也可在检测区进行多路分析，降低流体的损耗率，提高病原体检测效率和特异性以及检测结果的准确性^［23］^。

### 1.3 纸芯片制作

#### 1.3.1 蜡印法

蜡印法是通过打印机将固体墨水打印在纸上，进行图案绘制^［24］^，该制造方法简便、使用广泛。在纸的一面打印好图案后，加热使蜡穿透纸张^［25，26］^，在纸上形成疏水通道。Naorungroj等^［27］^通过蜡印法制备了一种基于比色法的μPAD，用于检测HPV。该法LOD为1 nmol/L，线性范围是1~10^3^ nmol/L，检测时间小于30 min。然而，蜡印法的性能在很大程度上受限于其分辨率，即经加热处理后形成的蜡屏障的最小宽度与精细度。尽管通过调整图案设计可适配不同微流控芯片需求，显示了一定的应用灵活性，但该技术仍难以满足高精度复杂图案的加工要求；此外，蜡印法操作流程虽简单，但优化分辨率所需的双面打印等额外操作，增加了操作复杂度；而商业化进程则因固体墨水打印机停产、技术精度不足等问题面临重大阻碍。

研究人员针对分辨率问题，开发了以下两种方法来提高分辨率，第一种是在纸的正面和背面都印上蜡，再用热层压法快速融化蜡，在双面形成固定的通道，避免单侧蜡印渗透而形成的通道分辨率低^［28］^，第二种方法是将纸浸入NaIO_4_，减小纸的表面积，使图案小型化^［29］^。蜡印法适用于基层医疗初步筛查等对精度要求较低的快速检测场景，但在技术突破分辨率瓶颈、解决设备供应问题并建立标准化流程前，其大规模商业化应用及更广泛的技术拓展将受到严重制约。

#### 1.3.2 光刻法

该法是一种微加工技术，能够制造出具有高精度的微通道（大约5 µm）。先用特定化学结构光刻胶浸纸，光刻胶在紫外线照射后发生化学变化，再将纸曝光于紫外线，光刻胶与纸发生聚合反应后，该区域变为疏水性质，进而根据掩膜模具的形状生成疏水通道，最后，用显影液去除未被曝光的光刻胶。光刻法以其高分辨率和良好的重复性在μPAD制备领域占据重要地位。然而，传统光刻法需掩膜模具，制约了生产效率并提高了成本，Park等^［30］^通过光刻法制备了一种基于Mie散射的μPAD，用于提高沙门氏菌（*Salmonella*）的检测特异性。该法可实现单细胞水平检测，线性范围为10~10^5 ^CFU/mL，检测时间小于1 min。近年来，科研人员不断探索创新，Zhang等^［31］^提出了一种无需掩膜的光刻技术，该技术工艺简便、芯片精度高、成本低、制作快速（曝光时间仅需2 s），该法克服了传统光刻法的局限性，为μPAD的制备提供了更高效、低成本的解决方案。该技术既保留了光刻法高精度的核心优势，又降低了对模具的依赖，在实际操作中简化了流程，显著提升了商业化潜力，有望推动光刻技术在微流控领域的大规模应用。

#### 1.3.3 喷墨打印法

喷墨打印可以根据需求快速设计芯片图案，并通过精确控制墨水沉积进行绘制^［32］^。相比于光刻法，该技术无需昂贵设备和复杂掩膜工艺，凭借低成本墨水和简化流程，尤其适合实验室和小规模生产^［33］^。此外，其能够在同一芯片上同时打印不同功能的墨水，实现多检测功能集成，提高芯片检测效率。然而，喷墨打印机精度低，在对微流控通道尺寸精度要求极高的应用中，影响检测的准确性，需进一步优化硬件措施，以满足更高精度要求。Deng等^［33］^的研究体现了该技术的应用特性，其通过喷墨打印法制备了一种荧光式µPAD，以3种荧光碳点为传感材料，将其与所选试剂混合，形成荧光墨水，再将荧光墨水和疏水墨水打印在滤纸上，实现多路荧光分析，其制备时间控制在10 min内，分辨率达到毫米级（2~10 mm），验证了喷墨打印法在POCT领域的应用潜力。喷墨打印法凭借低成本、易操作和多功能集成特性，在便携式检测、现场快速分析等场景中优势显著，但其精度短板仍需通过技术革新加以突破，以拓展至更高精度要求的应用领域。

#### 1.3.4 激光切割法

该法作为一种先进的微制造方法，能在基底材料上有效创建亲水、疏水区域，实现液体流动的精确控制^［34］^，并在纸芯片的制备中获得了广泛关注。激光切割法通过调整参数，可适配单层、多层或3D复杂微流控结构，还能与表面化学修饰等技术结合拓展功能维度；微米级切割精度和材料化学稳定性可保障微通道尺寸的一致性与检测的重复性；实际操作虽无需掩膜但需专业人员调试参数并结合流体力学设计结构。如Zhong等^［35］^使用激光切割和刀刻技术制备了µPAD，用于检测癌胚抗原，激光切割机在NC膜表面切割图案，形成疏水边界，再利用刀刻技术进行微沟槽加工，两种技术结合可以精准控制靶标溶液流动，扩大了µPAD的应用范围。该µPAD能够有效缩短试剂暴露时间并减少样品蒸发，从而促进抗原与抗体的充分结合，提高检测性能。其商业化前景广阔，核心优势包括高精度制造能力、可规模化生产以及适用于生物检测、化学分析和环境监测等多种场景。然而，较高的设备投入成本与材料兼容性挑战仍在一定程度上限制了该技术在中小型机构中的推广应用。

#### 1.3.5 激光打印法

先将墨粉打印在滤纸上，再进行热固化处理，形成疏水通道，该法制备的μPAD具有良好的柔韧性、高分辨率、可复制性、易生物降解性，被认为是制造μPAD的理想选择之一^［36，37］^。Ghosh等^［36］^用激光打印法制备比色式µPAD（LP-µPAD）检测*E. coli*（如图1）。其采用激光打印直接将碳粉油墨绘制形成图案，实现疏水性碳粉油墨的自动沉积，大大降低了制作成本和复杂性，总制备时间为20~30 min，其制备过程无需光刻、蜡印等专业设备，成本相较于传统方法降低50%。激光打印法凭借低成本、易操作和环保特性，在便携式检测和一次性芯片领域具有应用潜力，尤其适用于资源有限地区的现场快速分析。然而，若需拓展至高端医疗或工业检测领域，需进一步突破当前打印机分辨率限制、增强芯片在复杂环境中的稳定性，并通过技术创新降低核心设备依赖。

**图 1:**
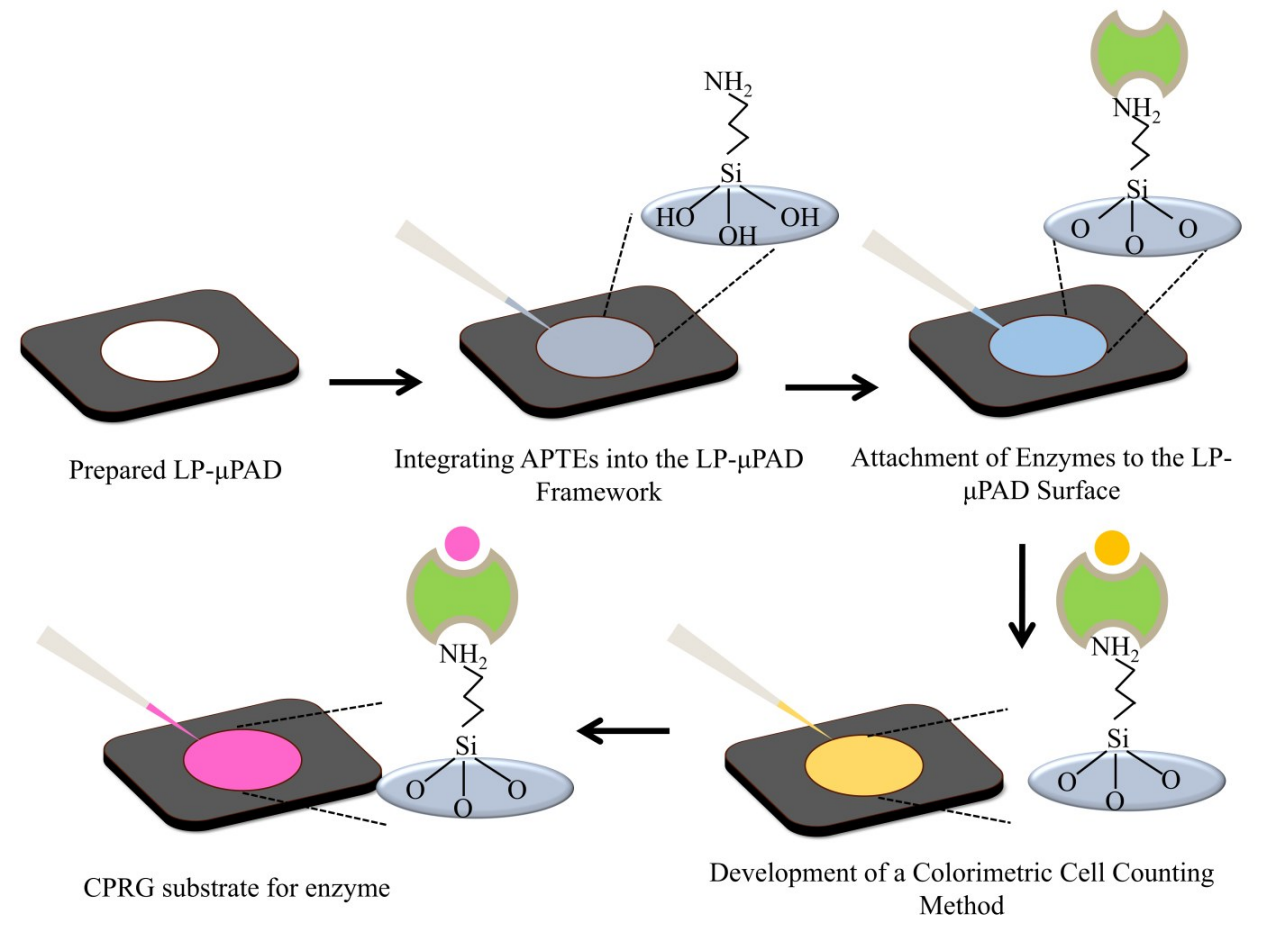
检测目标物中*E. coli*活细菌细胞的方案^［36］^

#### 1.3.6 热转移打印法

首先用热打印机打印碳带，在纸上绘制所需图案，而后进行烘烤，将蜡或墨粉融入纸表面，形成疏水屏障，冷却后用于实验。该方法保留了蜡印法的便利，产品性能与激光打印相当，在制作μPAD方面具有极大的潜力^［24］^。打印机的质量、打印材料的种类、碳带等因素都对产品产生影响，其中碳带的专一性、仪器的专业性等增加了制作成本和复杂度，目前这些劣势在很大程度上掩盖了其优势，限制了其商业化。上述方法优缺点如表2所示。除此以外，还有很多µPAD制作方法，如丝网印刷法、柔板印刷法等，但由于制作方式的专业性强、设备不便携、受环境影响大等原因，未实现广泛应用。

**表2:** **µ**PAD的制造工艺

Technology	Advantages	Disadvantages	Refs.
Wax printing	easy to manufacture and simple to operate	low-resolution， solid ink printer discontinued	［^25^，^26^，^28^，^29^，^38^，^39^］
Photolithography	rapid fabrication of μPAD with good precision	highly specialized， expensive equipment	［^5^，^40^-^42^］
Inkjet printing	The product is easy to operate， has a high resolution， and is cost-effective	low precision	［^32^，^33^，^43^］
Laser cutting	quick one-step production	expensive and highly specialized equipment	［^34^，^35^］
Laser printing	high precision	difficult pattern drawing， expensive instruments	［^36^，^37^，^44^］
Heat transfer printing	fast production， high-resolution， and durable µPAD	high production costs and complex assembly of carbon ribbons	［^24^，^45^］

## 2 检测方法

检测方法是影响设备整体性能的另一个重要因素，包括准确性、灵敏度、分析时间和分析成本。µPAD的检测方法主要包括比色法、电化学法、荧光法和化学发光法^［46］^。

### 2.1 比色法

将染料或酶标记的识别分子固定在试纸的检测区。当靶标分子被识别分子捕获时，产生的颜色变化可用于定性（通过肉眼观察）或定量（通过仪器如分光光度计测量颜色强度）分析。Huang等^［47］^建立了一种基于免疫分析法的三明治型LFA，可在45 min内检测人类多瘤病毒（BK virus，BKV）DNA。对于合成单链DNA的LOD为5 nmol/L。特异性研究显示与其他多瘤病毒无交叉反应性。对于含有BKV质粒的大肠杆菌培养样品，测定灵敏度为10^7^ copies/mL。该方法为在医疗点环境中检测各种目标分析物提供了巨大的潜力。比色法结果直观、判读简单，因此目前商品化的试纸多为比色式。尽管它们多为半定量分析，但在检测成本、速度、灵敏度、特异性等方面均表现优异。此外，该法与智能手机等智能设备结合，可以有效降低主观误差，实现精准定量分析。

### 2.2 电化学法

电化学法因其高灵敏度、强特异性、结果准确^［36，48-51］^的特点，满足了µPAD对检测性能的严格要求，因此近年来，与µPAD相结合的研究逐渐增多^［52］^，展现出显著的应用潜力。该法基于电极与待测物质之间的化学反应，通过测定电压、电流或电阻等信号的变化，实现对靶标分子的定性和定量检测，常见的电化学检测方法包括电位法、极谱与伏安分析法、方波伏安法等。Teengam等^［53］^研制了一种用于检测HPV的新型纸基电化学生物传感器，该传感器利用蒽醌标记吡咯烷基肽核酸探针（AQ-PNA）和石墨烯-聚苯胺（G-PAN）修饰电极。他们采用喷墨打印技术制备了纸基G-PANI修饰的工作电极，并通过静电吸附作用将负电AQ-PNA探针固定在电极表面。采用方波伏安法测定AQ标记杂交前后的电化学信号响应。该传感器在最佳条件下对HPV-DNA 16的LOD为2.3 nmol/L，线性范围为10~200 nmol/L。电化学法与µPAD结合具有多项优势，包括检测快速、成本低、便携性好、多功能集成、操作简单以及实时监测等。这些优势使电化学µPAD的应用与POCT的实际需求高度契合。

### 2.3 荧光法

荧光检测法具有较高的灵敏度。该方法将荧光标记的生物探针固定在检测区，当靶标分子存在时，通过检测荧光信号的强度变化进行定性或定量分析。其基本原理主要包括以下3种：直接荧光检测、荧光标记检测和荧光猝灭与恢复检测。Wang等^［54］^开发了一种基于荧光纳米颗粒标记抗体的LFA，用于快速检测SARS-CoV-2 RNA。该技术将荧光纳米颗粒标记于S9.6单克隆抗体上，当存在靶标RNA时，DNA探针与RNA先杂交，随后单克隆抗体捕获DNA-RNA杂交体，通过检测荧光信号强度，实现无扩增核酸检测，检测时间小于1 h。目前，商品化的荧光分子品类繁多，如绿色荧光蛋白（GFP）^［55］^、荧光素（fluorescein）^［56］^、罗丹明（rhodamine）^［57］^及Alexa Fluor染料^［58］^等。通过将抗体、DNA等生物分子与荧光燃料共价相连，可基于荧光标记检测原理，借助抗体或脱氧核酶与靶标分子的特异性结合，显著提高检测的特异性。

### 2.4 化学发光法

化学发光法是一种基于靶标分子与识别分子发生化学反应并产生光信号、通过对光信号强度进行测量以实现定性或定量分析的方法。该法无需外加光源，具有灵敏度高、设备简单、成本低、线性范围宽和重现性好等特点，因此在病原体检测等领域展现出巨大的应用潜力^［59，60］^。其有望实现自动化、并能够快速检测多种病原体。

综上所述，上述4种检测方法在µPAD中各有其适用场景：比色法适用于现场半定量筛查，电化学法擅长痕量核酸的检测，荧光法在检测DNA、RNA、抗原抗体等高特异性需求场景表现优异，而化学发光法则在自动化检测方面具备发展潜力。然而，这些方法仍存在一定的局限性，如比色法的定量精度有限、电化学法对电极材料和兼容性要求较高、荧光法依赖较高成本的检测设备、化学发光法则受反应稳定性的制约。未来的研究可聚焦于多检测方法集成、智能化设备的适配及低成本耗材的开发，从而进一步拓宽µPAD在高端医疗诊断和复杂环境现场检测中的应用范围。

## 3 **µ**PAD在病原体检测中的应用

### 3.1 细菌

细菌广泛分布在自然界，存在于水、土壤、空气等多种环境中。它们在人类环境中发挥着复杂的作用：在人体肠道中，部分细菌如双歧杆菌是益生菌，有助于维持肠道健康；而另一些有害细菌如金黄色葡萄球菌（*Staphylococcus aureus，S. aureus*），则会引发多种疾病；在土壤中，细菌能分解有机物、促进植物生长；在水体中，它们既可以净化水质、维持生态系统稳定，也可能通过破损皮肤感染宿主，创伤弧菌就是其中之一；空气中的细菌虽有助于维持生态平衡，但也可传播病原菌，如结核分枝杆菌（*Mycobacterium tuberculosis*，MTB）和*E. coli*等。由于细菌种类繁多，其对生态环境和人体健康的影响也具有显著差异。因此，研发能够对细菌进行高特异性、高灵敏度检测的POCT技术，已成为细菌相关疾病预防和控制领域的一个重要研究方向。

目前，有多种基于比色法的μPAD已被用于细菌的检测与分析。Rodriguez-Quijada等^［61］^研发了一种基于AuNPs的免疫LFA，用于直接检测创伤弧菌（*Vibrio parahaemolyticus*，Vp），将抗Vp抗体固定于NC膜上以捕获目标细菌；检测时，Vp与AuNPs偶联抗体形成“三明治”复合物并产生可见颜色信号，从而实现定性和定量分析，在优化条件下（抗体偶联浓度1.8 mg、硝酸纤维素膜CN140、0.1% BSA封闭），该LFA对Vp的LOD为4.66×10^5 ^CFU/mL，此值低于Vp致病感染剂量（ID_50_），表明该方法具备检测实际感染风险浓度的能力。Bu等^［62］^构建了一种LFA用于检测病原体。研究采用经半胱氨酸（Cys）和十六烷基三甲基溴化铵（CTAB）修饰的金纳米颗粒（分别为AuNPs@Cys和AuNPs@CTAB），使其与病原体结合形成复合物，此复合物在层析过程中被NC膜上固定的单克隆抗体捕获，并在T线上逐渐积累，导致T线颜色逐渐加深，通过测定发光强度可实现定量分析。该µPAD具有高的灵敏度和特异性，*E. coli* *O157*和肠炎沙门氏菌（*S. enteritidis*）结合AuNPs@Cys时，LOD分别为10^5 ^CFU/mL和10^3 ^CFU/mL，*E. coli O157*和*S. enteritidis*结合AuNPs@CTAB时，LOD皆为10^4 ^CFU/mL。Jokerst等^［63］^设计了一种μPAD，用于检测食品中大肠杆菌O157：H7、*Salmonella spp*及单核增生乳杆菌。该法检测原理基于目标病原体特异性酶与相应显色底物反应所产生的颜色变化来实现对致病菌的定性及半定量的分析。该µPAD对即食肉中的致病菌的检出限LOD低至10 CFU/cm^2^。Asif等^［64］^研发了一种用于检测*S. aureus*和*E. coli*及其耐药菌株的µPAD。其浸渍特异性显色底物后，*S. aureus*使其由无色变为淡紫色，*E. coli*则使其由黄色变为红紫色。在乳样品中，对*S. aureus*和*E. coli*及其耐药菌株的LOD均为10^6^ CFU/mL；牛奶样品经富集后，LOD低至10 CFU/mL，该µPAD基于多重显色机制，结合低成本与便携式设计，为食源性病原菌及其耐药性的现场快速筛查提供了一种高效解决方案，尤其适用于发展中国家的乳制品安全监测中的应用。Santopolo等^［65］^研发了一种比色µPAD，用于快速识别能够产生碳青霉烯酶和头孢菌素酶的病原体。该法在纸基上修饰带正电荷的聚二烯丙基二甲基氯化铵（PDDA），通过静电吸附作用捕获并富集表面带有负电荷的细菌，再将含特定抗生素及可选抑制剂/辅因子的溶液与纸基接触，以刺激病原体，当病原体产生*β*-内酰胺酶时，可水解抗生素，再以酚红pH指示剂，精准识别不同类型的*β*-内酰胺酶，进而判断细菌类型。该装置可用于诊断尿路感染，且有望发展为全自动诊断平台。

比色法可以通过DNA链间的杂交反应实现对细菌的检测。Trinh等^［66］^设计了一种比色式µPAD，用于筛查携带*vanA*和*vanB*基因的万古霉素耐药性肠球菌（Vancomycin-resistant Enterococcus，VRE）。该法在碱化的玻璃微纤维滤纸盘上进行DNA的提取和纯化，采用LAMP技术对靶标DNA进行扩增，并以酚酞作指示剂，实现了VRE的高灵敏度和高特异性检测，其LOD低至10^2^ CFU/mL，检测时间小于75 min。Fu等^［67］^研发了一种集核酸提取与扩增的μPAD，用于检测单核细胞增生李斯特菌（*Listeria monocytogenes*，Lm）。该μPAD通过功能化磁性硅珠对目标DNA进行分离，再进行PCR扩增，扩增后的产物与结合垫上的AuNPs-硫醇化DNA探针结合，其共轭产物在NC膜的检测区与DNA探针杂交产生信号，完成定性和定量分析，其对Lm的LOD低至10^4^ CFU/mL。该μPAD通过集成化设计、磁性分离增效，突破了传统分子诊断对专业设备的依赖，为食源性病原菌的POCT提供了高效解决方案，在食品安全、临床诊断等领域具有广泛应用潜力。

尽管比色法已展现出良好的检测效能，但随着技术迭代更新，部分学者开始探索灵敏度更高的荧光检测法。Morales-Narváez等^［68］^设计了一种基于免疫分析的LFA，实现了病原体的高灵敏度检测。该法在试纸条的T线和C线上分别固定带有抗体的量子点（Ab-QDs）荧光探针和裸露QDs。其检测原理基于氧化石墨烯（GO）的光致发光猝灭效应：当目标病原体存在，其与T线上的Ab-QDs 结合，阻碍GO与QDs的共振能量转移，使T线荧光信号得以保留，而C线始终被GO猝灭。通过计算T线与C线的荧光强度比值，即可实现对病原体的定量分析。在PBS缓冲液中，该LFA的LOD低至10 CFU/mL，在瓶装水、牛奶等复杂基质中，LOD也可达10^2^ CFU/mL。

同时，在追求高灵敏度细菌检测的探索道路上，电化学法也备受关注。Bhardwaj等^［69］^开发了一种基于电化学免疫的µPAD，用于检测*S. aureus*。该方法首先将检测抗体固定到单壁碳纳米管（SWCNTs）上，形成Ab-SWCNT共轭物，随后将其修饰在工作电极表面，当*S. aureus*与电极接触时，会引起电流响应发生变化，从而实现对细菌浓度的定量检测。表面增强拉曼散射（surface enhanced raman scattering，SERS）是一种光谱分析领域的信号增强技术，其具有高灵敏度、高分辨率、无需标记等优点，已被广泛应用于生物分析与检测领域。将比色法和SERS技术结合，可实现双重信号放大，进一步提高检测灵敏度与准确性。He等^［70］^介绍了一种基于铂涂层金纳米棒（AuNR@Pt）的双模式免疫LFA，用于检测空肠弯曲菌（*Campylobacter jejuni*，*C. jejuni*）。该法利用AuNR@Pt酶活性和SERS增强性能，实现了*C. jejuni*的双重检测，在SERS模式和比色模式下的检测范围分别为10^2^~ 5×10^6 ^CFU/mL和10^2^~10^6^ CFU/mL，LOD分别为75 CFU/mL和50 CFU/mL，该双模式LFA通过纳米材料功能整合与双信号校验，为病原体检测提供了一种灵敏度高、准确性好与经济实用的新工具，尤其适用于资源有限环境下的现场快速筛查。

Ma等^［71］^构建了基于功能DNA超结构（3D DNA）的荧光计数平台，用于蛋白质和microRNA的定量检测。该平台首先制备了具备高荧光性并携带针对目标物识别元件的3D DNA生物标签。检测时，目标物与表面固定的捕获抗体或DNA寡核苷酸以及3D DNA生物标签形成三明治复合物，随后通过荧光显微镜计数荧光点进行技术以实现定量分析。在以*β*-内酰胺酶和miR-21为检测对象的实验中，该方法LOD分别为100 amol/L和1 fmol/L，检测时长小于2 h。该平台优势在于检测时间短、可通过传统荧光显微镜分辨单个目标结合事件以及3D DNA生物标签功能多样且易于功能化。此研究可检测蛋白质和microRNA可作为病原体感染的生物标志物，为纸基微流控芯片检测病原体相关生物标志物提供了新策略，显著提升了检测的灵敏度和特异性。Chi等^［72］^开发了液滴DNAzyme偶联滚环扩增（dDRCA）系统，用于细菌检测。该系统以DNAzyme为细菌识别元件，通过微流控液滴封装实现数字化反应与定量，并利用滚环扩增（RCA）实现信号放大。在检测大肠杆菌时，将大肠杆菌裂解物、DNAzyme试剂及RCA试剂等乳化并封装于液滴中，若样本中存在大肠杆菌，DNAzyme将切割含RNA的DNA序列，引发RCA反应，扩增产物与荧光底物结合后可产生荧光信号，实现对靶标物的判别与定量。此系统能在1.5 h内完成对临床尿液样本中大肠杆菌的选择性检测，LOD低至单个细胞，并表现出良好的特异性。在复杂生物基质中检测性能稳定，已成功应用于尿路感染的临床诊断实践中。

目前，基于比色法的μPAD在细菌检测方面应用广泛。该类方法通过抗原抗体反应或DNA杂交等原理，可对多种细菌实现定性或定量分析。荧光检测法凭借其高灵敏度，可通过荧光信号的强度变化实现对细菌的检测。电化学法则通过监测细菌引起的电化学参数变化，实现对细菌浓度的定性与定量分析。近年来，通过将不同检测方法之间进行结合，各取所长，有效提升了细菌检测的灵敏度和准确性，也为POCT的细菌分析提供了可靠且高效的解决方案。

### 3.2 病毒

病毒作为一种广泛存在于人类环境中的微生物，对人类健康、公共卫生及社会经济等多个方面产生了深远的影响。病毒的持续传播和快速变异显著增加了疾病防控的难度，使得各国往往需要投入大量人力和物力资源以支持检测、治疗及防控工作。疫苗是目前预防病毒感染的首要措施，但病毒的快速变异可能导致免疫逃逸，这是疫苗开发中的重大挑战。为有效应对病毒疫情，研究人员正不断研发兼具低成本、快速、操作简单、高灵敏度与高特异性的检测技术，以期实现病毒的高效POCT。

病毒肆虐使科研人员意识到，发展精准高效的检测技术对疫情防控工作至关重要。Teengam等^［73］^研发了比色式μPAD，用于检测中东呼吸综合征冠状病毒（MERS-CoV）、MTB和HPV的DNA。该芯片采用折纸法制作3D芯片，实现上下分层检测，显著提高了检测通量。该芯片对3种病毒的DNA均表现出高灵敏度，LOD分别为1.53 nmol/L（MERS-CoV）、1.27 nmol/L（MTB）和1.03 nmol/L（HPV）。Yen等^［74］^介绍了一种基于多色银纳米颗粒（AgNPs）的LFA，可用于区分DENV、黄热病和埃博拉病毒。该方法将不同AgNPs与特定抗体结合形成AgNP-Ab复合探针。当含有病毒蛋白的靶标溶液加到样本垫中，其与AgNP-Ab结合形成AgNP-Ab/抗原复合物，并依据T线上呈现的不同颜色实现病毒的视觉区分。该装置灵敏度高，对3种病原体的LOD均为150 ng/mL。Biswas等^［75］^制作的μPAD，集捕获、纯化、洗脱于一体，用于检测DENVRNA，该芯片可同时检测4种DENV，操作简单，特异性达100%，LOD低至5 copies/μL**。**该μPAD实现全流程集成与多血清型同步检测，为全球DENV高流行区提供了一种低成本、高特异性且操作简便的现场筛查工具，尤其适用于基层医疗点和疫情应急响应等场景。McCracken等^［76］^介绍了一种使用免疫凝集颗粒流变学传感技术的µPAD，用于快速检测病原体。该µPAD采用蜡打印技术在纸上构建疏水通道，通过监测免疫凝集反应引起的样本流变学特征变化，实现对抗体与靶标分子相互作用的定量分析。该μPAD灵敏度高、受环境影响小，对水样中*E. coli K12*及生物基质中ZIKV的LOD为100 CFU/mL和0.53 copy/mL。Mazzu-Nascimento等^［38］^制作了一种免疫式µPAD，用于检测犬瘟热病毒（Canine Distemper Virus，CDV）。该装置在纸表面创建疏水通道，并通过物理吸附方式将兔抗CDV抗体固定在AuNPs表面形成免疫探针，当含有CDV的样品溶液通过时，会发生特异性抗原-抗体反应，在T线处形成可见的红色线条，从而实现对CDV的视觉判读。

荧光法凭借其高灵敏度和高特异性，不仅在细菌POCT中应用广泛，在病毒检测领域也表现出显著优势^［33，77-79］^。Weng等^［80］^研发了一种基于荧光共振能量转移式的µPAD，用于诺如病毒的快速、高灵敏度检测。该方法首先使用多壁纳米管（MWCNT）或GO作为荧光猝灭剂，将6-FAM-适配体的荧光猝灭，当检测到诺如病毒后，适配体与MWCNT或GO分离，恢复荧光，两种方法的LOD分别为4.4 ng/mL和3.3 ng/mL。Yuan等^［81］^介绍了一种μPAD可同时检测铜绿假单胞细菌（*Pseudomonas aeruginosa，P. aeruginosa*）和*S. aureus*。该研究将石墨烯量子点（graphene quantum dots，GQDs）和金纳米簇（gold nanoclusters，AuNCs）分别与*S. aureus*蛋白A抗体和*P. aeruginosa*外毒素A抗体进行偶联。当目标抗原存在时，会与相应偶联物结合，GQDs和AuNCs经过UV照射发出特征荧光，从而实现对快速、高灵敏度定性及定量分析。该µPAD检测时间仅需10 min，对于蛋白A和外毒素A的LOD分别为0.2 ng/mL和0.1 ng/mL。Brunauer等^［82］^介绍了一种基于核酸免疫分析LFA，用于直接检测病原体的DNA。该方法将病原体粗裂解液直接进行重组酶聚合酶扩增（recombinase polymerase amplification，RPA），产生双标记扩增产物，随后将扩增产物加入试纸条中，并利用羊抗地高辛抗体标记的荧光微球来检测双标记扩增子，总耗时小于30 min。在以*P. aeruginosa*进行评估中，该法的LOD为2.1×10^5 ^CFU/mL，且无交叉反应。荧光检测法在μPAD中的成功应用，为POCT、环境监测等领域提供了高效、低成本的解决方案。

随着检测技术的不断发展，化学发光法已成为μPAD检测方法的新起之秀。Jin等^［83］^研发一种基于化学发光的μPAD，通过检测ATP实现对病原体的间接检测。该芯片采用蜡打印技术在纸上构建疏水通道，在μPAD上固定ATP的适配体，进行杂交等操作，通过Z折叠驱动液体运输，在H_2_O_2_催化下，3-氨基-9-乙基咔唑（AEC）发生氧化并产生显色信号，从而指示检测结果。研究团队验证了该μPAD可检测*Salmonella*，其对ATP和*Salmonella*的LOD分别为1 μmol/L，2.6×10^7^ CFU/mL。该μPAD基于ATP的通用检测机制，适用于多种微生物的筛查，尤其适用于资源有限地区的食品卫生筛查和疫情初筛，为全球公共卫生监测提供了一种经济、高效的检测工具。未来可通过优化适配体亲和力或集成多重检测通道，进一步提升该方法的特异性与检测通量。

结核病（tuberculosis，TB）是由MTB引起的一种传染性疾病，已成为全球范围内尤其是发展中国家的重大公共卫生问题，造成了极高的死亡率和社会经济负担^［84］^。Tung等^［85］^设计了一种基于DNA纳米酶的比色μPAD，用于检测MTB。该方法从MTB中选取特异性基因片段作为靶标DNA，并将其互补序列分为捕获DNA和报告DNA（sDNA）。将捕获DNA修饰到T线上，sDNA修饰在钯铂双金属纳米颗粒（Pd@PtNPs）的表面。当样品溶液中存在MTB，靶标DNA可与捕获DNA及Pd@PtNPs-sDNA探针结合，进而触发Pd@PtNPs的纳米酶活性，催化TMB生成蓝色的ox-TMB，从而实现可视化检测，检测时间小于15 min，其LOD低至0.22 nmol/L。该方法结合了金属纳米酶的催化特性和分子杂交技术，简化预处理过程，降低检测成本、提高检测效率。

丙型肝炎病毒（Hepatitis C Virus，HCV）是一种常见的血源性病原体，感染后会导致肝炎，目前尚无针对HCV的有效疫苗，尽管直接抗病毒药物的出现使得预防和治疗丙型肝炎提供了可能，但其并未得到普及。因此，开发高效、低成本的HCV检测方法尤为重要。Ozefe等^［86］^研发了一种基于免疫吸附的µPAD，用于HCV检测。该µPAD固定了HCV NS3蛋白的抗体，接着依次加入HRP偶联的抗山羊IgG/HRP抗体和TMB。当存在HCV时，即可在检测区产生蓝色信号。在PBS缓冲体系中，肉眼观察、智能手机和显微镜分析的LOD分别为1、0.88和0.80 ng/mL；而在人血浆中，肉眼和智能手机的LOD分别为10 ng/mL、2.20 ng/mL。与传统ELISA相比，该平台具有制备简单、成本低、检测灵敏等优点，适用于资源有限条件下的HCV筛查。

流感是一种由流感病毒引起的急性呼吸道传染病，根据核蛋白和基质蛋白的差异，可分为甲型（A）、乙型（B）、丙型（C）和丁型（D）4种类型，其中甲型（Flu A）和乙型（Flu B）是导致人类流感的主要病原体。Wu等^［87］^设计了一种纸基点ELISA系统，用于检测Flu A。该装置由储存区和反应区组成，反应区的T线和C线分别固定14B11抗体和另一种特异性酶捕获抗体。当存在Flu A抗原时，其被14B11抗体捕获后，随后加入HRP，T线和C线均呈紫色；阴性对照仅C线变紫色；失败测试则C线空白。为了提高灵敏度，该团队还设计并比较了两种孵育腔形状，I型底层有两个小孔，Ⅱ型有一个圆通孔。结果表明，I型中样品缓慢流经NC膜，抗原、抗体和底物反应充分，信号更强，检测准确性和灵敏度高。Lei等^［88］^设计了一种无酶夹心免疫分析LFA，用于检测和区分Flu A的H1N1和H3N2亚型。该LFA将抗H1和抗H3抗体固定在T线上。当加入病毒样本后，再与鼠抗IgG-AuNP发生特异性结合，再经金增强技术实现信号放大，随后根据特异性信号判断病毒类型。该法仅用5 μL试剂，约1 h即可完成检测，操作简便、快速，与常规ELISA相比有明显优势。

研究人员积极探索，将多种检测方法与μPAD进行结合，以满足多种病毒检测的需求。这些成果为病毒的早期发现、精准防控提供了坚实的技术支撑，有力推动了生物化学检测领域在病毒防控方面的发展。

## 4 总结与展望

µPAD凭借低成本、易加工、可生物降解等优势，在病原体诊断领域显示出巨大的应用潜力。当前，以Whatman滤纸和NC膜为代表的纤维素基材料是制备µPAD芯片的主要材料，其良好的生物兼容性与化学稳定性为芯片的整体性能提供了重要保障。在结构设计上，µPAD的结构类型主要分为2D和3D两种类型。3D芯片是在2D结构基础上通过折叠、弯曲等工艺扩充了其空间架构，不仅实现了多路检测功能，也显著提升了检测效率与特异性。在µPAD的制作方法方面，呈现出多样化的特点，常见的技术包括蜡印法、光刻法、喷墨打印法、激光切割法、激光打印法以及热转移打印法等，可根据不同应用场景灵活选择。

目前µPAD的研究方向主要聚焦于4个方面，一是新材料的研发，旨在进一步提高检测的灵敏度与特异性；二是对µPAD结构的优化与制作工艺的升级，力求设计具备多功能的µPAD，以实现对多种类型病原体的同步检测或在芯片上的多步操作；三是检测方法的创新改进，例如与色谱、拉曼光谱、电化学等技术的联合应用，增强检测性能；四是推动µPAD的商业化与应用普及，使其在病原体检测等领域得到更为广泛的普及。值得注意的是，随着大数据计算与人工智能等前沿技术的深度融合，µPAD有望彻底革新病原体检测模式，真正实现快速、便捷、经济的即时诊断与生化分析技术的跨越式发展，为医疗健康领域带来前所未有的变革与突破。
